# Initiation and Maintenance of Low‐Dose Transdermal Testosterone in Non‐Binary Individuals: A Retrospective Audit

**DOI:** 10.1111/cen.70110

**Published:** 2026-02-22

**Authors:** Eli Ward‐Smith, Ada S. Cheung, Peter Locke, Sav Zwickl, Brendan J. Nolan

**Affiliations:** ^1^ Trans Health Research Group The University of Melbourne Melbourne Australia; ^2^ Department of Endocrinology Austin Health Melbourne Australia; ^3^ Equinox Gender Diverse Clinic, Thorne Harbour Health, Abbotsford Victoria Australia

**Keywords:** gender identity, non‐binary, testosterone, transdermal, transgender

## Abstract

**Objective:**

An increasing number of trans individuals, particularly those who are non‐binary, desire lower testosterone doses than those outlined in current guidelines for gender affirmation. These guidelines acknowledge the need for a tailored approach to treatment, especially for non‐binary people. However, existing guidelines for initiation of testosterone assume that trans individuals desire rapid and complete masculinisation through standard dosing. We aimed to assess the initiation and maintenance of low‐dose testosterone in non‐binary individuals treated with transdermal testosterone for ≥ 6 months.

**Design:**

Retrospective audit.

**Patients:**

Non‐binary individuals initiating low‐dose transdermal testosterone with ≥ 6 months follow‐up.

**Intervention:**

Testosterone 1% gel (< 50 mg daily) or testosterone 5% cream (< 100 mg daily).

**Measurements:**

Transdermal testosterone dose and serum total testosterone concentration.

**Results:**

Forty‐six individuals were included. Median age was 27 years (24−30) and duration of testosterone was 14 months (9−24). For individuals treated with testosterone 1% gel, median dose at initiation was 12.5 mg (12.5−25) and 25 mg (20.4−37.5) at last follow‐up (*p* < 0.01), and for those treated with testosterone 5% cream, the median dose at initiation was 50 mg (31.3−50) and 50 mg (50−68.8) at last follow‐up (*p* = 0.02). Median serum total testosterone concentration was 11 nmol/L (5.2−15.7). By the last follow‐up, 40 (87%) remained on low‐dose testosterone and 6 (13%) had increased to full‐dose testosterone. In a subgroup of 30 individuals with ≥ 12 months treatment, 26 (87%) remained on low‐dose testosterone by last follow‐up. Three (7%) individuals had polycythemia (haematocrit > 0.5).

**Conclusions:**

Most non‐binary individuals initiating low‐dose transdermal testosterone continue doses lower than those recommended in current guidelines after 6−12 months of treatment. These hypothesis‐generating findings highlight the need for studies evaluating the influence of low‐dose testosterone on clinical outcomes and safety end‐points.

## 1 Introduction

Testosterone therapy is a necessary component of gender affirmation for some trans and gender‐diverse (trans) individuals to permit the development of physical characteristics to align with their gender identity. Non‐binary people are individuals whose gender does not fit exclusively into the woman/man binary and make up an estimated 26%‐40% of the adult trans community [1, 2, 3]. Notably, not all non‐binary people identify under the broader ‘trans umbrella’ [4].

Historically, gender affirming treatment guidelines were based on binary notions of gender and, as a result, non‐binary people have experienced barriers to accessing gender affirming healthcare [5, 6]. These factors have also contributed to a perception that non‐binary people rarely desire or pursue gender affirming hormone therapy (GAHT) [3, 4]. However, recent data have indicated that GAHT is a priority for many non‐binary people, with rising numbers seeking access to GAHT [3].

In trans people recorded female at birth who desire it, testosterone GAHT is associated with improvements in mental health and quality of life [7, 8, 9, 10], with a randomised clinical trial demonstrating clinically significant improvements in depression, gender dysphoria and suicidality with early access to conventional full‐dose testosterone [7].

Current consensus guidelines for gender affirmation give recommendations for standard doses and formulations of testosterone treatment that have typically been used for hypogonadal cisgender men [5, 11]. The World Professional Association for Transgender Health (WPATH) Standards of Care Version 8 (SOC 8) comprehensively discusses the importance of adopting a personalised and collaborative approach to the provision of gender affirming care for non‐binary people [5]. However, an increasing number of trans individuals, including those with a non‐binary gender identity, express a desire for lower testosterone doses than those recommended in these guidelines to induce slower or lesser degrees of masculinisation [12, 13]. However, there is a lack of evidence to inform the definition and prescription of ‘low‐dose' testosterone, and the existing consensus guidelines do not provide such guidance [5, 11, 14].

In Australia, transdermal testosterone formulations include testosterone 1% gel or testosterone 5% cream. These formulations have been shown to be bioequivalent (50 mg testosterone 1% gel is bioequivalent to 100 mg testosterone 5% cream) [15], and both are recommended in the Australian position statement on the hormonal management of adult trans and gender diverse individuals [14]. Testosterone 1% gel (Testogel, Besins Healthcare, Sydney, Australia) is most prescribed as a pump actuation bottle, where each pump actuation delivers 12.5 mg testosterone and the recommended standard starting dose is 50 mg daily [14]. Testosterone gel at doses of 50−100 mg daily is also recommended in the Endocrine Society guidelines [11]. Testosterone 5% cream (AndroForte 5, 5%w/v (50 mg/mL); Lawley Pharmaceuticals, West Leederville, Australia) is a non‐alcohol‐based cream with a recommended standard starting dose of 2 mL (100 mg) of cream by measured applicator per day when applied to the upper body (torso, abdomen and sides of the body) [14]. Transdermal testosterone may be a favourable option for those seeking low‐dose testosterone therapy due to the ability to titrate the dosage as desired embodiment goals evolve [13, 16].

There are limited data evaluating low‐dose testosterone regimens, tailored to the embodiment goals expressed by non‐binary people [13, 16]. Previous data have demonstrated that a higher proportion of people with a non‐binary gender identity receive tailored GAHT regimens [17]. Similarly, previous retrospective audits have demonstrated high rates of low‐dose transdermal testosterone use in non‐binary individuals [12, 13], but no study has evaluated longitudinal prescription of low‐dose transdermal testosterone.

With the increasing visibility of non‐binary identities and an increasing number of non‐binary people seeking GAHT in recent years, there is an urgent need for greater awareness and understanding of the unique gender affirming healthcare needs for non‐binary people [3, 5]. As such, in this retrospective study of non‐binary individuals initiating low‐dose transdermal testosterone, we aimed to assess the maintenance of low‐dose testosterone after 6‐12 months.

## 2 Materials and Methods

A retrospective audit of electronic medical records was performed of consultations of trans and gender‐diverse individuals at a primary care clinic and an endocrine clinic in Melbourne, Victoria, Australia. Data were collected from new consultations between 22 February 2016 and 12 September 2021. The audit was approved by the Austin Health Human Research Ethics Committee (Audit/21/Austin/69) and Thorne Harbour Health Community Research Endorsement Panel (THH_2024_022), and the nature of the study did not require informed consent.

This retrospective audit included non‐binary individuals who initiated low‐dose transdermal testosterone and had accurate documentation of testosterone dose and serum total testosterone concentration after ≥ 6 months of treatment. Treatment decisions were guided by clinical response and collaborative approaches to decision‐making between individuals and clinicians, as per the national guidelines [14]. Transdermal testosterone formulations included 1% testosterone gel (Testogel, Besins Healthcare, Sydney, Australia) and 5% testosterone cream (AndroForte 5, 5%w/v (50 mg/mL); Lawley Pharmaceuticals, West Leederville, Australia). Individuals with a binary gender identity, commencement of conventional full‐dose transdermal testosterone, or < 6 months of testosterone treatment were excluded.

The primary outcomes of interest were serum total testosterone concentration and transdermal testosterone dose after ≥ 6 months of treatment. Testosterone doses were classified as low dose if less than those recommended in Australian consensus guidelines (< 50 mg for testosterone 1% gel and < 100 mg testosterone 5% cream). We also analysed the prevalence of polycythemia. Polycythemia was defined as haematocrit > 0.5, as described in the Endocrine Society clinical practice guidelines of gender‐dysphoric/gender‐incongruent persons [11].

Gender identity was obtained from intake forms and medical records. Classification of binary and/or non‐binary gender identity was as previously described [18]. In short, binary gender identity was defined as identification as exclusively male (including trans male, trans man, and transgender male), and non‐binary gender identity encompassed all other identities (including non‐binary, transmasculine, genderqueer, and agender). If an individual reported more than one gender identity, of which one was categorised as non‐binary, they were categorised as having a non‐binary gender identity for the purpose of data analysis.

As data were obtained retrospectively, total testosterone concentrations were measured using immunoassays available as standard care in Australia. All laboratories were accredited by the National Association of Testing Authorities (NATA, the national accreditation body for Australia). Serum total testosterone concentrations are typically measured at baseline, every 3 months during the first year of testosterone treatment, and then every 6−12 months once stable [14].

Statistical analyses were performed using STATA version 17.0 software (StataCorp. 2021. *Stata Statistical Software: Release 17*. College Station, TX: StataCorp LLC). Data were not normally distributed so median (IQR) are reported. The Wilcoxon signed‐rank test was used to compare the testosterone 1% gel and testosterone 5% cream doses at baseline and last follow‐up. Spearman's rank correlation coefficient was used to assess the correlation between testosterone dose and serum total testosterone concentration. *p* < 0.05 was considered statistically significant.

## 3 Results

**Figure 1:**
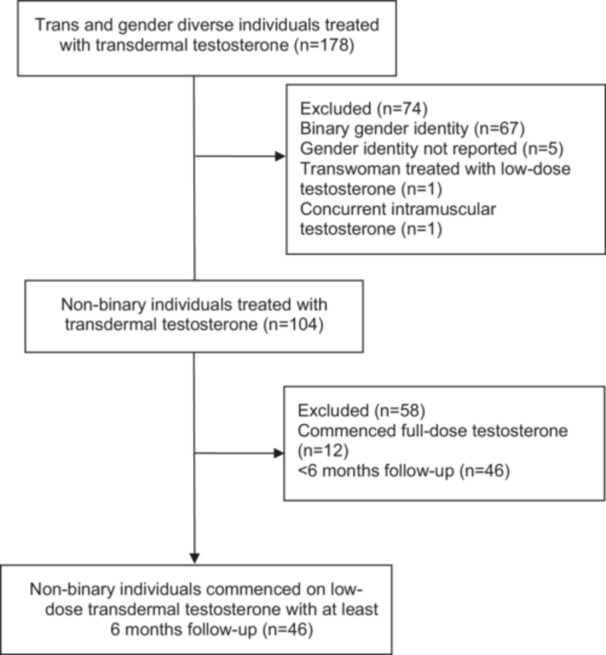
Cohort selection strategy.

Data were collected from 178 trans and gender‐diverse individuals prescribed transdermal testosterone, of whom 104 (58%) had a non‐binary gender identity. Of these, 12 individuals commenced full‐dose testosterone and 46 had less than 6 months follow‐up, leaving 46 individuals for analysis (Figure 1).

Median age was 27 years (24−30) and median duration of testosterone at last follow‐up was 14 months (9−24) (Table 1). Twenty‐four (52%) individuals were treated with testosterone 1% gel and 22 (48%) were treated with testosterone 5% cream.

**Table 1:** Baseline characteristics.

	Testosterone 1% gel (*n* = 24)	Testosterone 5% cream (*n* = 22)	Overall cohort (*n* = 46)
Age (years)	28 (25–30)	25 (23–29)	27 (24–30)
Duration of GAHT (months)	19 (11–29)	11 (8–21)	14 (9–24)
Baseline serum total testosterone (nmol/L)	1.0 (0.9–1.4)	1.2 (0.8–1.6)	1.1 (0.8–1.5)
Initiation dose (mg)^a^	12.5 (12.5–25)	50 (31.3–50)	25 (12.5–50)

*Note:* Data are presented as median (interquartile range)

^a^ Conventional full‐dose of testosterone 1% gel is 50 mg daily and testosterone 5% cream is 100 mg daily

**Figure 2:**
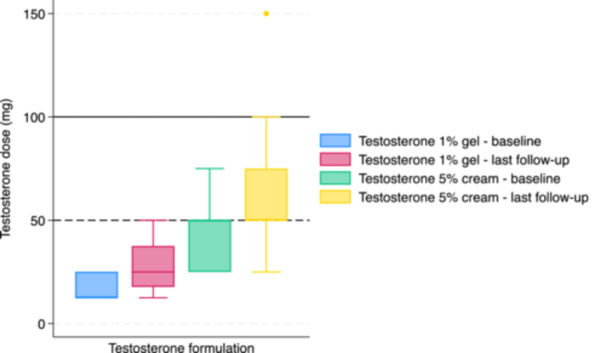
Dose of testosterone 1% gel and testosterone 5% cream at baseline and last follow‐up. The dotted horizontal line indicates the conventional full‐dose for testosterone 1% gel (50 mg daily) and the solid horizontal line indicates the conventional full‐dose for testosterone 5% cream (100 mg daily).

**Figure 3:**
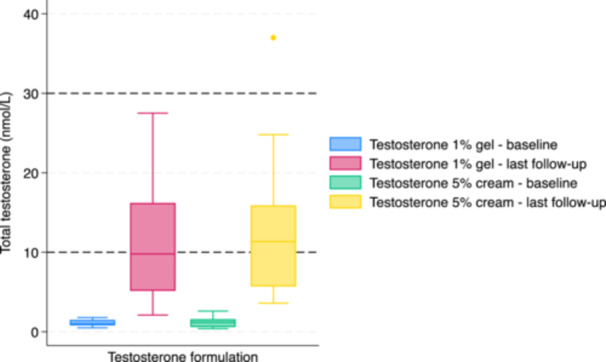
Serum total testosterone concentration at baseline and at last follow‐up with testosterone 1% gel and testosterone 5% cream. Dotted horizontal lines indicate the cisgender male reference range of 10‐30 nmol/L.

For individuals treated with testosterone 1% gel, the median dose at initiation was 12.5 mg (12.5−25) and 25 mg (20.4−37.5) at last follow‐up (*p* < 0.01) (Figure 2). By the last follow‐up, 20 (83%) remained on low‐dose testosterone and 4 (17%) had increased to full‐dose. Median serum total testosterone concentration at last follow‐up was 9.8 nmol/L (5.2−15.6) (Figure 3). Thirteen (54%) had serum total testosterone concentrations < 10 nmol/L at last follow‐up.

For individuals treated with testosterone 5% cream, the median dose at initiation was 50 mg (31.3−50) and 50 mg (50−68.8) at last follow‐up (*p* = 0.02) (Figure 2). By the last follow‐up, 20 (91%) remained on low‐dose testosterone and 4 (17%) had increased to full‐dose. Median serum total testosterone concentration at last follow‐up was 11.35 nmol/L (6.0−15.3) (Figure 3). Nine (41%) had serum total testosterone concentrations < 10 nmol/L at last follow‐up.

In the overall cohort, 40 (87%) remained on low‐dose testosterone and 6 (13%) had increased to full‐dose testosterone. In a subgroup of 30 individuals with ≥ 12 months of testosterone treatment, 26 (87%) remained on low‐dose testosterone and 4 (13%) had increased to full‐dose testosterone at last follow‐up. In a subgroup of 21 individuals with serum testosterone concentration < 10 nmol/L on the first follow‐up bloods, of whom 15 had subsequent bloods, 11 (73%) individuals remained on low‐dose testosterone and 4 (27%) increased to full‐dose.

The median serum total testosterone concentration was 11 nmol/L (5.2−15.7) at last follow‐up. In all, 23 (50%) were in range 10−30 nmol/L, with 22 (48%) < 10 nmol/L and 1 (2%) > 30 nmol/L. There was no correlation between testosterone 1% gel or testosterone 5% cream dose and serum total testosterone concentration (*r* = 0.20, *p* = 0.29 and *r* = 0.11, *p* = 0.51, respectively).

Three (7%) individuals had polycythemia (haematocrit > 0.5) during treatment with low‐dose testosterone; all individuals were treated with testosterone 1% gel with doses 12.5−25 mg and serum total testosterone concentrations 5.6−39.6 nmol/L (2 readings < 10 nmol/L).

## 4 Discussion

In this retrospective cohort study of non‐binary individuals commencing low‐dose transdermal testosterone, the majority continued low‐dose testosterone at the last follow‐up. Median serum total testosterone concentration was at the lower end of the cisgender male reference range.

### 4.1 Comparison to Previous Literature

Despite previous misconceptions that non‐binary people do not pursue medical intervention, more recent data now demonstrate that a significant proportion desire GAHT [3, 19]. In a cohort of 218 people recorded female at birth from Australia, 106 (48.8%) reported current use of GAHT, 19 (8.8%) reported previous but not current use of GAHT, and 12 (5.5%) were taking steps to commence GAHT [3]. The GAHT dose or route of administration was not reported in this online survey. Another recent online survey reported lower rates of GAHT use in non‐binary people recorded female at birth compared to trans men, but the reasons and individual treatment desires were not provided [20]. Doses and route of administration were not reported.

There has been limited data evaluating hormone regimens in non‐binary people [4, 16]. A retrospective analysis from a specialist gender clinic in the Netherlands from 2013 to 2015 reported that individuals with a non‐binary gender identity were less likely to receive conventional GAHT (82% vs. 92%, *p* = 0.004) and were more likely to be prescribed tailored GAHT (11% vs. 4.7%, *p* = 0.02) than trans men and trans women. Of the 273 (45%) trans individuals recorded female at birth who were treated at the clinic, there was no difference in serum testosterone concentrations between trans men and non‐binary individuals. However, it is unclear what testosterone doses were prescribed, though the authors described that ‘tailored’ GAHT could include adjusted or lower testosterone doses [17].

The physical health outcomes for trans men receiving testosterone GAHT, and the impact of ‘low’ versus ‘high’ testosterone doses were investigated at a specialist gender clinic in Japan [21]. Body composition changes and laboratory parameters were measured, and patients were stratified according to the mean testosterone dosage received in the first year. Most patients received testosterone enanthate, with ‘low‐dose' defined as ≤ 62.5 mg/week, and ‘high‐dose’ defined as > 62.5 mg/week. Of the 291 patients included, 188 (64.6%) received low‐dose testosterone. Although the low‐dose cohort had lower serum testosterone concentrations, it should be noted that the mean total testosterone concentration remained in the cis male range consistently for both cohorts in the first 12 months.

A review of hormone initiation in Aotearoa, New Zealand found that over a third of patients presenting for GAHT to a primary care clinic between 2020 and 2022 were non‐binary. Eighteen (56%) people recorded female at birth had a non‐binary gender identity, of whom 5 (28%) requested low‐dose testosterone [22].

### 4.2 Embodiment Goals

Although this analysis has focused on non‐binary people, it is important to acknowledge that embodiment goals from testosterone should not be conflated with one's gender identity [4, 16]. Testosterone is typically understood as a ‘male’ hormone, tied to masculinity and particular body parts which are ‘binary gendered’. However, for non‐binary patients seeking GAHT, it may have a very different role in assisting a shift ‘away’ from their sex recorded at birth and markers of womanhood/femininity, rather than ‘moving towards’ manhood and masculinity [3, 23].

Similarly, some non‐binary people desire full‐dose testosterone GAHT. For example, in a randomised trial evaluating early access to conventional full‐dose testosterone therapy, 48% reported a non‐binary gender identity [7]. Conversely, some trans men may desire low‐dose testosterone therapy. Conceptualising desired physical characteristics as ‘embodiment goals’ might help individuals commencing low‐dose testosterone to visualise a future body that feels in alignment with their gender identity [24]. Such goals may also provide the non‐binary person and their practitioner with a framework for discussing their desired physical changes, and how these might be achieved [25]. These goals may also provide the person with expanded capacity for self‐expression as their physical characteristics align with their gender identity [24]. Ultimately, clinicians should take a personalised approach to the provision of GAHT, which is individualised to the person's embodiment goals and titrated according to clinical response [14]. These findings may suggest that non‐binary people intentionally make requests for low‐dose testosterone, in alignment with embodiment goals.

### 4.3 Limitations

Limitations to this analysis are related to its retrospective design, including missing data that were not collected in a standardised manner and a lack of data regarding dispensing and treatment adherence. Body mass index (BMI) and ethnicity were not consistently documented in the medical records. Specifically, verifiable weight data were missing for a number of patients during the COVID pandemic, when clinics transitioned quickly to telehealth to facilitate continuity of care. Therefore, we are not able to determine if BMI influences serum testosterone concentration.

Serum total testosterone concentration results were collected via different immunoassays available for routine clinical care and not collected at a standardised time in relation to the last administered dose. Liquid chromatography‐mass spectrometry (LC‐MS) is considered the reference standard for sex steroid measurement, but is not available in routine clinical care in Australia [26]. Luteinizing hormone (LH) and follicle‐stimulating hormone (FSH) were not collected as standard care and therefore not available for analysis.

Prescriber preference and the rationale for testosterone formulation and/or dose were not consistently documented. It is also possible for the 3 individuals who developed polycythaemia, clinical decision‐making relating to testosterone dosage may have been impacted. We also did not have data on patient satisfaction, masculinising clinical effects, and physical outcomes such as cardiovascular risk profiles, dysphoria, or quality of life.

Of our eligible cohort, a large number had follow‐up for < 6 months, so were excluded from this analysis. Patients were classified as having a binary or non‐binary gender identity by the researchers, based on intake forms and medical records. We also have not evaluated low‐dose testosterone use in people with a binary gender identity. Finally, the testosterone formulations used in Australia differ from those in other jurisdictions, in which standard ‘full‐dose’ can be less than 50 mg daily (our definition of ‘low‐dose’ testosterone).

### 4.4 Clinical Implications and Future Directions

The majority of non‐binary people continued testosterone 1% gel and testosterone 5% cream at doses below the recommended range for hypogonadal cisgender men. Notably, the median serum total testosterone concentration was at the lower end of the cisgender range, and there was significant inter‐individual variability, so there is consideration for lower doses should an individual desire slow or lesser degrees of masculinisation. In this context, the lowest starting doses of 12.5 mg testosterone 1% gel or 25 mg testosterone 5% cream could be considered.

Prospective trials evaluating clinical and mental health outcomes in individuals initiating low‐dose testosterone treatment are required. Such studies could evaluate clinical outcomes such as body composition or voice deepening, laboratory measures including sex steroid concentrations, bone density, cardiovascular risk and adverse effects such as polycythemia, with a comparator group of individuals commencing standard full‐dose testosterone.

## 5 Conclusion

The majority of non‐binary individuals initiating low‐dose testosterone continued testosterone doses lower than those recommended in consensus guidelines after 6−12 months. Half of the individuals had serum testosterone concentrations within the cisgender male reference range, so there is consideration for initiation of the lowest dose (12.5 mg testosterone 1% gel or 25 mg testosterone 5% cream) in people seeking low‐dose testosterone. The findings of this descriptive, retrospective audit are hypothesis‐generating and highlight the need for prospective studies evaluating clinical and safety endpoints.

## Disclosure

Brendan J. Nolan and Ada S. Cheung have received product from Besins Healthcare for separate investigator‐initiated clinical studies using estradiol and progesterone. No monetary support from Besins Healthcare has been received for any studies and Besins Healthcare have had no input into study design, data analysis or writing of any manuscripts.

## Conflicts of Interest

The authors declare no conflicts of interest.
